# An Efficient Framework for Securing the Smart City Communication Networks

**DOI:** 10.3390/s22083053

**Published:** 2022-04-15

**Authors:** Faisal Abdulaziz Alfouzan, Kyounggon Kim, Nouf M. Alzahrani

**Affiliations:** 1 Department of Forensic Sciences, College of Criminal Justice, Naif Arab University for Security Sciences, Riyadh 14812, Saudi Arabia; 2 Center of Excellence in Cybercrime and Digital Forensics, College of Criminal Justice, Naif Arab University for Security Sciences, Riyadh 14812, Saudi Arabia; kkim@nauss.edu.sa; 3 Information Technology Department, Collage of Computer Science and Information Technology, Al Baha University, Al Bahah 65731, Saudi Arabia; noufalzahrani@bu.edu.sa

**Keywords:** smart city, cyber security for smart cities, communication wireless network, man-in-the-middle (MITM) attack, network intrusion detection system (NIDS)

## Abstract

Recently, smart cities have increasingly been experiencing an evolution to improve the lifestyle of citizens and society. These emerge from the innovation of information and communication technologies (ICT) which are able to create a new economic and social opportunities. However, there are several challenges regarding our security and expectation of privacy. People are already involved and interconnected by using smart phones and other appliances. In many cities, smart energy meters, smart devices, and security appliances have recently been standardized. Full connectivity between public venues, homes, cares, and some other social systems are on their way to be applied, which are known as Internet of Things. In this paper, we aim to enhance the performance of security in smart city communication networks by using a new framework and scheme that provide an authentication and high confidentiality of data. The smart city system can achieve mutual authentication and establish the shared session key schemes between smart meters and the control center in order to secure a two-way communication channel. In our extensive simulation, we investigated and evaluated the security performance of the smart city communication network with and without our proposed scheme in terms of throughput, latency, load, and traffic received packet per seconds. Furthermore, we implemented and applied a man-in-the-middle (MITM) attack and network intrusion detection system (NIDS) in our proposed technique to validate and measure the security requirements maintaining the constrained resources.

## 1. Introduction

The rapid rise of information and communication technology (ICT) has changed not only modern society, but also the automation system in industries, including the electric power system, to a more convenient and dependable system. Electric power system reliability is one of our society’s most basic necessities, and in today’s technologically oriented world, an efficient and dependable electric power system is heavily reliant on ICT [1,2]. In recent years, there has been a lot of interest in upgrading traditional power systems to smart grid systems, which has boosted the integration of power systems with ICTs to ensure a dependable system that can overcome the issues that traditional power systems face. Increased consumption is one of the issues, as is the incorporation of cutting-edge technologies into power networks, such as renewable energy generation, electric vehicle charging, and smart meters. The ever-increasing reliance on electricity, as well as the demand for high-quality power, have necessitated smarter power delivery, more accessible pricing, and faster power restoration [3].

Although a new city that connects everything through the internet smartly and electrically does not exist yet, many cities are on their way to develop the connectivity to be fully smart. However, the increasing number of security concerns have demonstrated that cyber attacks in smart cities are ubiquitous and can occur at any time. For critical infrastructure, complex cyber attacks can occur that may paralyze industrial control systems, which results in serious damage and terrible consequences. For humans, smart wearables, such as mobile ransomware and communications hijacking, are able to steal users’ data and personal identity information (PII). For society, changing or manipulation of these data can consequently lead to widespread panic and public opinion that can also cause threaten social management. Furthermore, the cyber security risks have significantly become the most serious threats in terms of developing the interconnect facilities in smart cities and the self-driven car industry, where accident liability will also become a particularly important and hot topic [4].

Therefore, communication trust and security challenges in the deployment of smart city systems have recently become a source of concern. In particular, a smart electrical meter distributed in multiple hierarchical networks can significantly achieve mutual authentication and establish the exchange keys. In order to enhance the performance of the smart city communication network, the OPNET modeler will be used to implement both (1) the secure scenario with authentication and key session techniques and (2) another scenario without these techniques. Various objectives should be processed and carried out in order to build our contribution. These include studying and investigating the security methods of authentication and confidentiality data relating to smart city technology and the impact of its integration with various emerging technologies, as well as selecting and designing authentication and a key exchange schemes approach in order to secure smart city data. In turn, this will include collecting data and conducting analysis of a secure control network in smart city communication, comparing and determining secure and insecure traffic parameters using authentication and key session schemes, and eventually evaluating the performance of a secure control network in smart city communication using authentication and key session schemes.

In this paper, we provide a new framework/scheme for enhancing secure control in smart city communication networks by boosting security performance. This works by enforcing data secrecy and authentication using efficient key exchange mechanisms. In order to secure a two-way communication channel, a smart city system can achieve mutual authentication and build shared session key schemes between smart meters and the control center.

The remainder of this paper is organised as follows. In Section 2, we review the related work. Section 3 describes our proposed cyber security framework for smart cities in detail. Section 4 presents and discusses the results of our simulation study. Finally, Section 5 concludes the paper.

## 2. Related Work

This section depicts the relevant background information and materials for this investigation. It will be described how to secure a smart city communication network as well as information and communication technology (ICT). This is followed by a literature review that focuses on reviews related to the security networks in smart city communication by implementing authentication and key exchange schemes. In addition, certain security strategies that can be applied as security requirements in smart city communication networks will be discussed in detail.

### 2.1. Securing Smart City Communication Network

Smart cities have piqued the interest of numerous disciplines, including scholars, enterprises, and governments, as a result of the rapid growth of ICT. Because a lack of proper cyber security can result in the theft of a user’s sensitive data, utility fraud, and grid instability, cyber security is a major concern in the implementation and adoption of smart cities [5]. The United States’ National Institute of Standards and Technology (NIST) established three critical needs for smart city security. These requirements are based on information availability, integrity, and confidentiality [6].

### 2.2. Information and Communication Technology (ICT) in a Smart City

**Figure 1:**
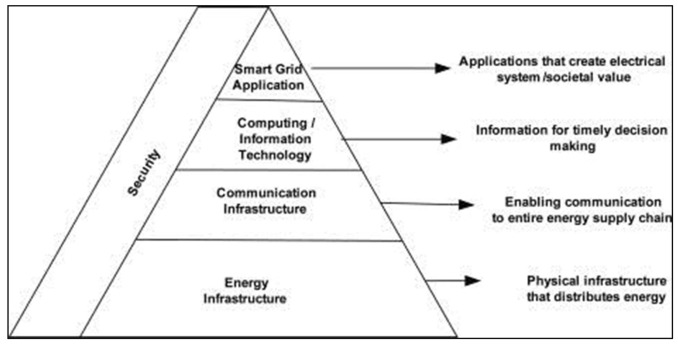
Presents the smart city definition.

As presented in Figure 1, the smart city system has been divided into four layers, each with its own set of needs in order to be more efficient, reliable, and intelligent. Furthermore, efficiency, reliability, scalability, and intelligence have become increasingly vital in order to be fast, better, secure, and resilient controls and communication. The deployment of ICT in smart grid has involved different applications such as advanced metering infrastructure, wide area measurement system, substation automation system, and common information models [7].

In a nutshell, overall, the primary purpose of a smart city is to focus on urban residents, not just to meet current requirements but also to ensure that future generations are kept in mind with regards to cultural, social, economic, and environmental aspects.

### 2.3. Offensive Cyber Security Framework

**Figure 2:**
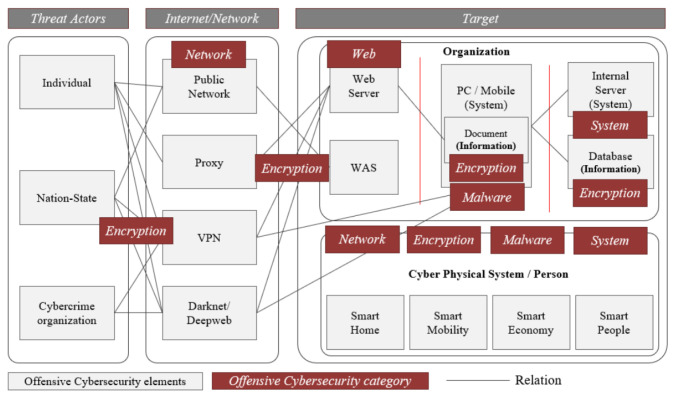
Offensive cyber security framework [8].

To examine the goal and flow of cyber attacks, we systematized aspects of the offensive cyber security framework. Individual hackers, cyber crime organizations, and nation-state hackers, as indicated in Figure 2, perform cyber attacks to achieve their goals, such as financial gain or system destruction. The framework is designed depending on the internet/network, treat actors, and targets. Moreover, individuals, nation states, and cyber crime organizations are all dangerous players in cyber attacks. In order to gain access to the attack target, the Internet and network are employed, with Public Networks, Proxies, Virtual Private Networks (VPNs), and the Darknet/Deepweb being used. Organizations and CPS are the most common targets of an assault.

There are open to the outside Web Servers and Web Application Servers (WAS) within the firm, as well as personal computers and mobile phones with documents and information stored on them. Internal systems and databases carrying critical information are also used by businesses. CPS and Persons have both been targeted in recent attacks. Smart Homes, Smart Mobility, Smart Economy, and Smart People are among the CPS’s detailed attack targets [9,10]. The assault categories that cyber attackers must breach, such as encryption, networks, web, malware, and systems, are referred to as offensive cyber security [8].

### 2.4. Literature Review

The integration of information and communication technologies in numerous application domains, such as transportation, academics, industries, medicine, and energy, has a significantly positive impact on society; however, systems’ inability to cope with vulnerable attacks and loss of confidentiality, integrity, and authenticity has stymied this integration [11]. All application domains with communication infrastructure are vulnerable to cyber attacks, with the smart city being the most serious and vulnerable. Sensing, communication, control, and actuation systems work together to enable bi-directional pervasive communication for a variety of applications, including monitoring real-time energy consumption, providing real-time information to consumers, smart monitoring and tracking, control system commands, and more. One of the most essential roles of SCADA systems for remotely monitoring the grid’s physical processes is power state system estimation [12]. The power system model and telemetered data help provide the best potential state of the system and can aid in ensuring appropriate power station performance through various applications. Because the system’s state is based on a power state system estimation, a value that is invalid, corrupt, or deceptive as a result of a vulnerable attack might cause the system’s state, management, and analysis to be diverted. Due to the risk to business-critical information, the bi-directional flow of information has generated a number of security and privacy issues; as a result, an efficient strategy is necessary to reduce complexity, computational cost, and processing time for immediate communication [13]. Although a large amount of research has been conducted in the realm of traditional cyber security, many approaches do not effectively meet the security requirements of grid systems. The following are some of the restrictions:

- Lifespan of grid systems;
- Proprietary system’s dependency;
- Remotely located resources;
- Lack of physical protection;
- Limited computational resources.

The need of key management has been acknowledged in relation to advanced metering infrastructure, but no plan to combat threats has been proposed [14]. In the context of AMI, a key management system (KMS) for unicast, multicast, and broadcast transmission has been developed to improve efficiency, key storage, computation, and administration of keys with forward and backward security based on key graphs [15]. The authors have taken into account key creation and key freshness, authentication and integrity, and forward and backward security, but they have left out the process of key distribution, key destruction, key renewal/revocation, and the node replacement phase. Furthermore, because storage costs have become a concern, SELINDA has been proposed as a key establishment and data collection protocol to allow power operators to initiate shared keys with various measurement devices through an untrusted data relaying data collector unit [16]. In order to assure security during data collection, the data collector unit was not deemed to hold any key established between the power operator and measuring devices. Because the power operator controls all public keys for all measuring devices, unauthorized access or a little breach can put the entire system at risk. Furthermore, untrusted data replay nodes can expose data to a man-in-the-middle attack. The authors of [17] investigated a trust anchor so that a data collector and a device may do mutual authentication and key establishment. The suggested technique considers man-in-the-middle and replay attacks and is based on the public key and Needham–Schroeder authentication protocol. The key benefit of this method is that it reflects high levels of security, fault tolerance, and accessibility [18].

However, the complexity of resource constraint nodes in a smart grid is great due to the integration of both PKI and trustworthy anchors. The approach is not scalable because as the number of devices grows, the trust anchor’s ability to perform mutual authentication and key generation may be limited. The authors of [13] demonstrated that a key management scheme for unicast, multicast, and broadcast has been discussed using the Iolus framework, which is based on a secure distribution tree using group security controllers (GSC) to manage top level subgroups and group security intermediaries to manage the remaining subgroups. The GSC is in charge of all subgroup keys, whereas subgroups are in charge of all node keys. In terms of scalability, the Iolus framework can be advantageous because changes in membership within a group will not affect other subgroups. The Iolus-based technique offers limited multicasting functionality as well as the production of a larger number of keys stored in remote terminal units, but at the cost of higher computational overhead and complexity [19].

From the above literature review, it should be emphasized that one of the most basic requirements for grid systems to enable appropriate resilience to attacks is an improved security system. Smart grid systems are expected to use a variety of authentication and secure key management mechanisms. Most of these secure key management systems and practices, on the other hand, are either incomplete or do not correspond to real-world scenarios and emerging technologies. As a result, it is crucial to understand that security requirements differ from one system to the next. As a result, in terms of secrecy, a planned design is required. However, [20] estimated that the D-H key exchange algorithm provides security for unprotected channels by exchanging secret keys over unprotected channels, whereas [21] argued that because the traffic between smart meters and utilities in the smart grid system is predicted to be massive, the most secure authentication and encryption solutions may not be the fastest. As a result, it is apparent that secure authentication and session key exchange systems have an impact on the smart grid communication network’s performance. Therefore, the goal of this paper is to enhance the performance of security in smart city communication networks by using a new framework and scheme that provide an authentication and high confidentiality of data by implementing them along with session key exchange schemes through the OPNET simulator modeler.

## 3. Description of Our Proposed Cyber Security Framework for Smart Cities

This section briefly gives an overview of our proposed framework followed by a description of each operational stage in detail. We finally analyze and evaluate our proposed framework by using a cyber attack and intrusion detection system.

### 3.1. Overview

The purpose of our proposed cyber security framework is to enhance the communication efficiency as well as to reduce any negative impacts of security. In other words, we aim to improve the security system, which is one of the fundamental requirements for smart city systems, and also to ensure the sufficient resilience between two parties without attacks. Notably, the security requirements can vary from system to system. Hence, the planned design is required in order to overcome these challenges at an early stage.

To deploy our proposed framework along with the key exchange Algorithm 1, two different tasks are used; namely, authentication and encryption between every smart meter and the control center in order to create a two-way secure communication.
**Algorithm 1:** Modifying the process of D-H keys exchange algorithm.
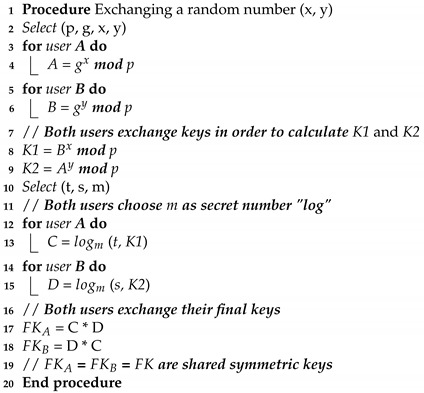


### 3.2. Secure Two-Way Communication by Using an Authentication and Key Exchange Scheme

**Figure 3:**
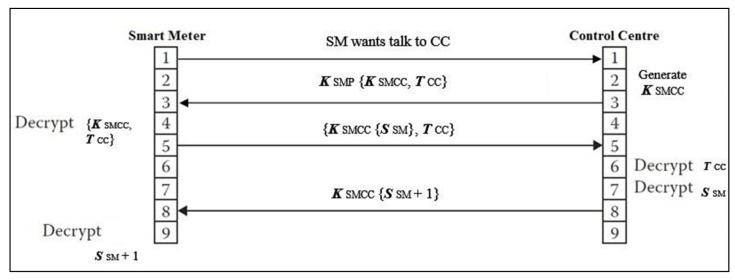
Secure two-way communication by providing an authentication and key session algorithm.

The authentication and session key discovery portion of secure two-way communication work is presented in Figure 3. Every message, *m*, will be authenticated and encrypted with key *K*. Now, every smart meter transmits *m* to the control center. This *m* is a trusted communication, which requests a session key for its communication with the control center. A control center generates a $K_{SMP}$, which includes a smart meter’s public key $K_{SMCC}$ and a ticket generated by the control center to secure the communication between them $T_{CC}$. Upon receiving the $K_{SMP}$, a smart meter immediately decrypts it. Another message, $K_{SMCC}$, is sent by the smart meter to the control center, which is a key session between them. This message contains $T_{CC}$ and random secret number $S_{SM}$. When the message is received, the control center decrypts both $T_{CC}$ and $S_{SM}$; then, it sends the message back to the smart meter as $S_{SM}$ + 1, which is a value of that message encrypted between them in order to protect the two-way communication.

For the authentication and key exchange scheme, which is used between a smart meter and the control center to securely provide a two-way communication as outlined above, two tasks configurations, authentication and encryption tasks, are illustrated below in detail.

### 3.3. Authentication and Encryption Tasks

The authentication task includes two phases, which are described as follows. In the first phase, a smart meter sends a single message with a specific size to the control center. None of the smart meters spend any initialization time to generate this message. In order to send a response message for every individual smart meter’s request, the control center spends only 0.5 s of processing time. The second phase involves confirming the authentication process in each smart meter, and it takes 0.2 s to process the control center’s return message.

During the second task, encryption, of securing two-way communication between each smart meter and the control center, two phases are applied to achieve the processing deployment. The first phase consists of encrypting messages before delivering them individually from each smart meter to the control center in order to ensure the connection between these two parties. These encrypted messages are uniformly distributed within a size of 1 to 10 kilobytes, and the process of the encrypting takes only 0.2 s. The second phase encrypts messages before sending them from the control center to each smart meter, as well as ensuring secure connection between them. Table 1 shows the authentication and encryption tasks, as well as the configurations for each phase, each of which has two phases that drive traffic from each smart meter to the control center, including two-way communication, authentication, and encryption.

### 3.4. Key Exchange Scheme

A cryptographic key exchange system is a method of exchanging cryptographic keys. We modified the (D-H) key exchange technique, which enables two-way communication between transmitters with no prior knowledge of one another to join and construct a shared secret key across unsecured network communications. As a result, using a symmetric key, this key may be used to encrypt subsequent transmission, which the destination can then decrypt to safely receive communications. The collaboration of Hitfield Diffie and Martin Hellman produced the first practical technique for establishing shared secret keys between two-side communications (such as A and B) via an unsecured communication network, as shown in our Algorithm 1. The modification of the D-H keys exchange technique is illustrated in the Algorithm 1. In this pseudocode, ‘*p*’ denotes a prime number and ‘*g*’ indicates a primitive root such that *g* < *p*. As ‘*g*’ is a primitive root of ‘*p*’, the numbers *g mod p*, $g^{2}$ *mod p*, …., $g^{x}$ *mod p* will produce all numbers from 1 to p−1. A and B are denoted as Alice and Bob, respectively, and *K1* and *K2* indicated the secret key of each. They select the arbitrary numbers ‘t’ and ‘s’ such that 0 < t, s < *p*. Now, the first user selects a random number (*x*) as its private key. Its public key is calculated as $g^{x}$ *mod p*. Similarly, the second user user selects a random number (*y*) as its private key. Its public key is calculated as $g^{y}$ *mod p*. The public keys are exchanged over a public channel in order to calculate *K1* and *K2*. Both users are now required to choose *m* as a secret number. Thereafter, both users exchange their final keys, and then shared the symmetric keys.

### 3.5. Cyber Attack Evaluation

In this subsection, the analysis and evaluation of our proposed framework requires first to know the nature of the cyber attack. Because of this nature, malicious malware incorporates increasingly complex strategies. As a result, we undertook a more in-depth study of the harmful activities while computing the cyber attack score. The 12 phases of the MITRE ATT&CK were subjected to an analysis, and the findings are displayed.

**Figure 4:**
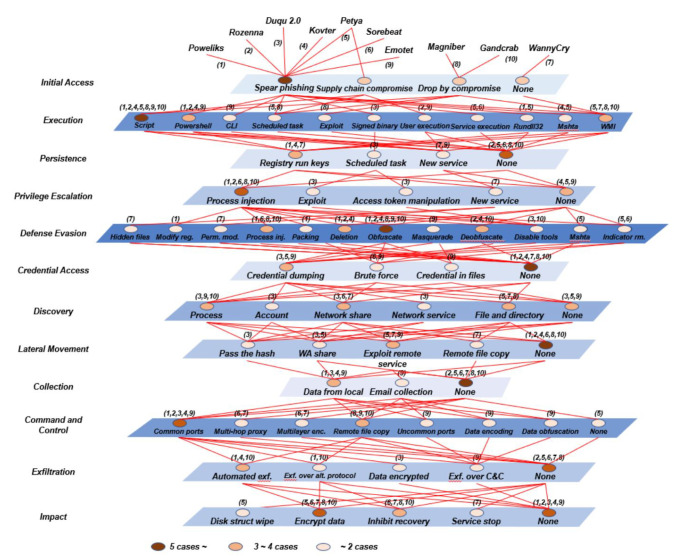
Attack techniques of a fileless cyber attack [8].

Each layer, in Figure 4, depicts the steps of a cyber attack in MITRE ATT&CK. The flow of an attack is determined by the layer order. The red line demonstrates a link between a malicious code technique and the previous approaches used. The color of the blue plane indicates the cyber attack strategies at each level. Furthermore, the circle’s hue shows the number of cyber attacks that have used it. For example, if there are five or more cyber attack kinds, the circle is dark brown; if there are three or four, it is orange; and if there are two or fewer, it is apricot [8,22,23].

Due to these potential cyber attacks that may occur in smart city communication networks, we implemented and applied a man-in-the-middle (MITM) attack and network intrusion detection system (NIDS) in our proposed technique to validate and measure the security requirements maintaining the constrained resources.

Signature-based systems (SBS) and anomaly-based systems (ABS) are examples of NIDS systems that can dynamically monitor and analyze system events to identify whether they are assaults or authorized accesses [24]. The goal of NIDS is to detect malicious behavior related to messages sent across the smart city communication network between a smart meter and the control center. Different sorts of assaults, such as replay and man-in-the-middle attacks, can be detected using the simultaneous network intrusion detection monitoring method. The given topology network is used to investigate the performance of the smart city wireless communication network by implementing authentication and session key exchange schemes, and also by preventing the man-in-the-middle attacks in order to measure our proposed security concerns over the smart city system.

**Figure 5:**
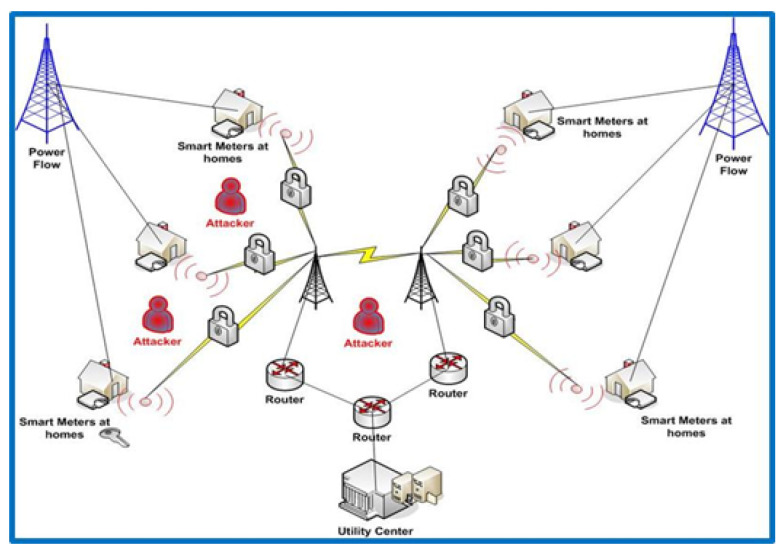
Implementing and applying a man-in-the-middle (MITM) attack and network intrusion detection system (NIDS) in our topology.

**Figure 6:**
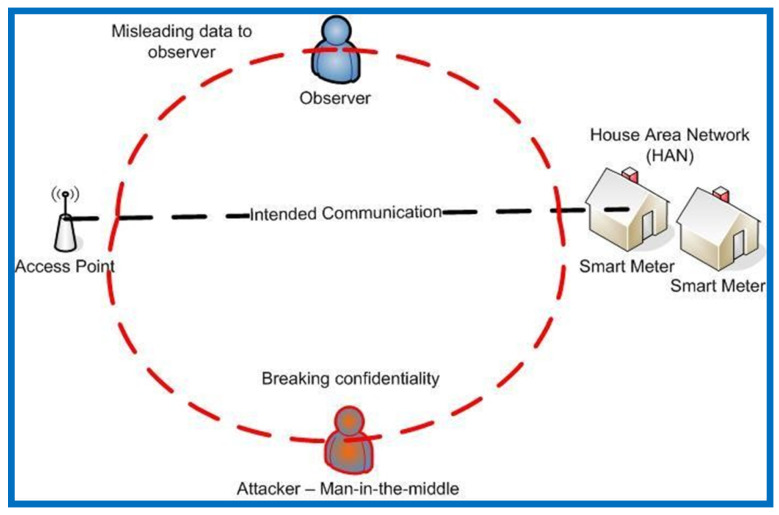
How a man-in-the-middle attack intercepted messages passing via the smart city communication network.

As seen in Figure 5, an MITM attack is characterized as follows: suppose entity “A” wants to monitor the messages sent by entity “B” as a smart meter to entity “C” as the control center server. A man-in-the-middle attack using keys can be conducted in this case to intercept messages between entities “B” and “C”. Thereafter, the interceptor can replace the message and deceive entity “A”. Entity “A” can be a mobile node observing data between a smart meter and a capturing device in the context of grid computing. Eavesdropping, replay attacks, and physical jamming attacks can all be treated in the same way as man-in-the-middle attacks. Consequently, Figure 6 shows how a man-in-the-middle attack can be detected and messages intercepted across the smart city communication network.

## 4. Simulation Network and Setup

In this section, we first describe our used simulation tool called OPNET. We then discuss the simulation setup of our proposed framework for enhancing the security control in smart city communication networks. We also use the OPNET simulator to study the performance of the smart city communication network with and without authentication and key exchange mechanisms. Various performance measures are defined in this study such as throughput (packet per seconds), delay (packet per seconds), load, and traffic received (packet per seconds). We finally present and analyze the simulation evaluation.

### 4.1. Network Simulator

A network simulator is a program that simulates networks with a variety of nodes. To create real-time network experiments, simulation tools are practically required. Several simulation tools, such as the OPNET modeler, and other open source simulators, such as NS-2, GNS2, GNS3, and OMNET, are available.

One of the most widely used data network modeling and network simulation technologies is the OPNET modeler. It is a comprehensive network modeling tool with a slew of useful features. The OPNET modeler, in particular, allows for the simulation of heterogeneous networks using various scenarios, as well as the simulation of specifically planned network architecture and analysis in order to compare different types of traffic and situations [25]. It is also used to support a variety of operating systems, including Linux and Microsoft. Because of its real-time configuration and operation capabilities, the fundamental advantage of using OPNET is the ability to evaluate and analyze networks in realistic experiments. As a result, OPNET allows for the comparison of network improvements based on multiple protocols, as well as the development of new protocols and technologies. OPNET also supports the following simulation technologies: Discreet Event Simulator (DES), Flow Analysis Simulation, ACE Quick Predict Simulation, and Hybrid Simulation [26]. Therefore, DES will be the simulation technology used in this study.

As shown in Table 2, we use the following parameters for both authentication and encryption tasks. Each has two phases, as mentioned above. In the first phase of the authentication task (Smart Meter to Control Center), we set up the requests to be in initialization time = 0 second, request count = 1 (constant), request packet size = 3 Kbytes (constant). For the response of this phase, the processing time = 0.5 s, number of replies = 1 (constant), request packet size = 5 kbytes (constant). In the second phase of the authentication task (Verify Control Center), we also set the requests in the initialization time = 0.2 s, request count = 0 (constant), whereas there is no response in this phase.

For the second task (encryption), the first phase (Smart Meter to Control Center) includes the following in the request field: initialization time = 0.2 s, request count = 1000 (constant), inter-request time = 1.2 s (exponential), request packet size = 1–10 kbytes (uniform_int). The request of the second phase (Control Centre to Smart Meter) is exactly the same as that in the first phase of the encryption task. Now, both requests in both phases have no responses.

All the results are averaged over 50 runs for randomly generated topologies, while the simulation time for each run is set to 3600 s.

### 4.2. Simulation Evaluation

**Figure 7:**
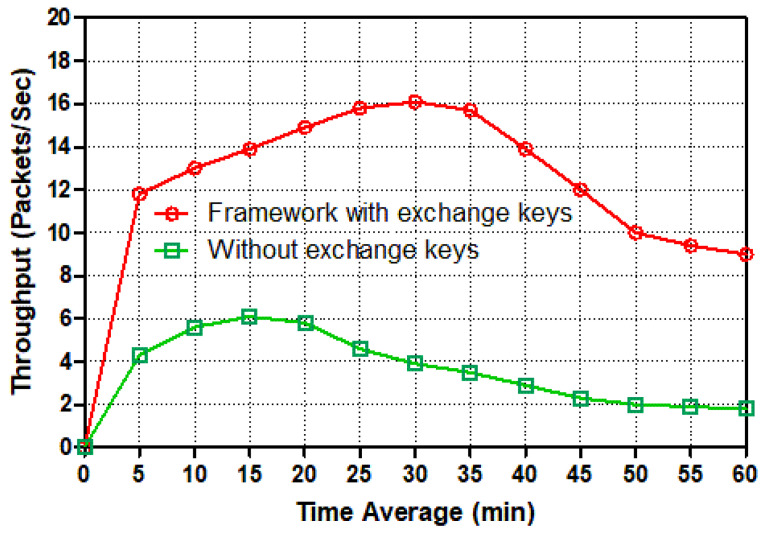
Throughput (packets/s) from a smart meter to the switch for different tasks.

**Figure 8:**
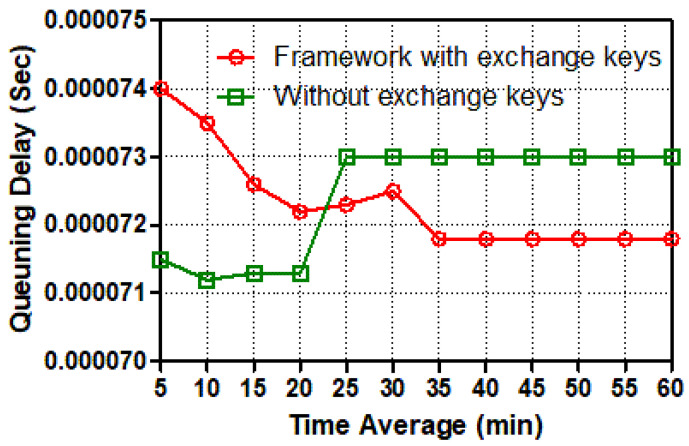
Queuing delay (s) from a smart meter to the switch for different tasks.

**Figure 9:**
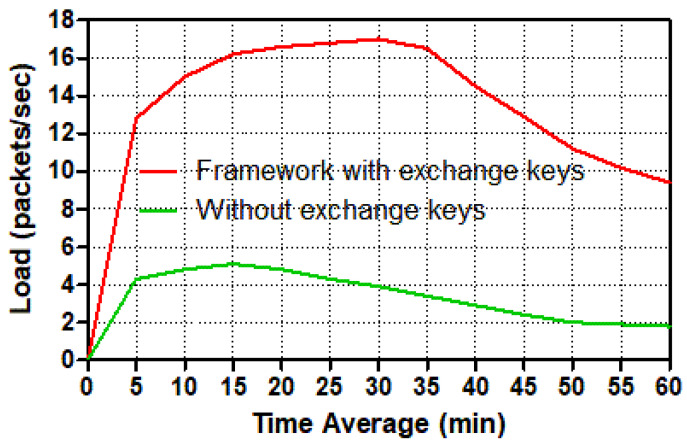
Load (packets/s) in a smart meter for different tasks.

**Figure 10:**
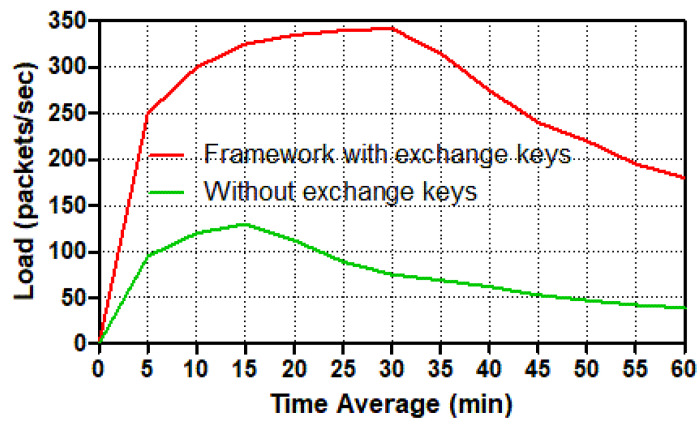
Load (packets/s) in the control center for different tasks.

**Figure 11:**
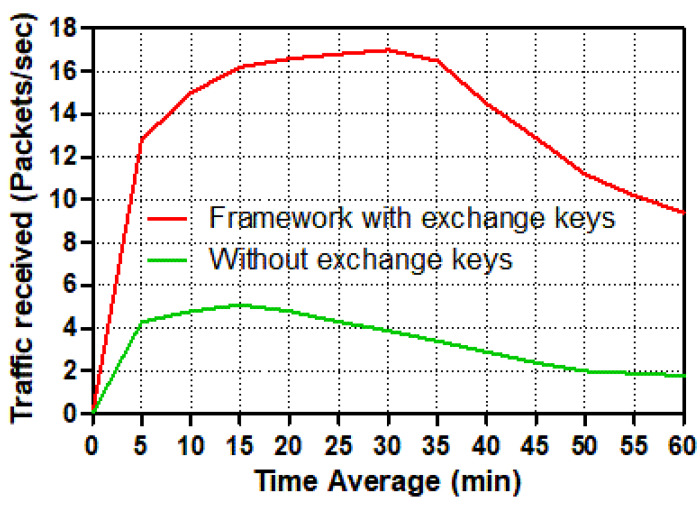
Traffic received (packets/s) in a smart meter for different tasks.

**Figure 12:**
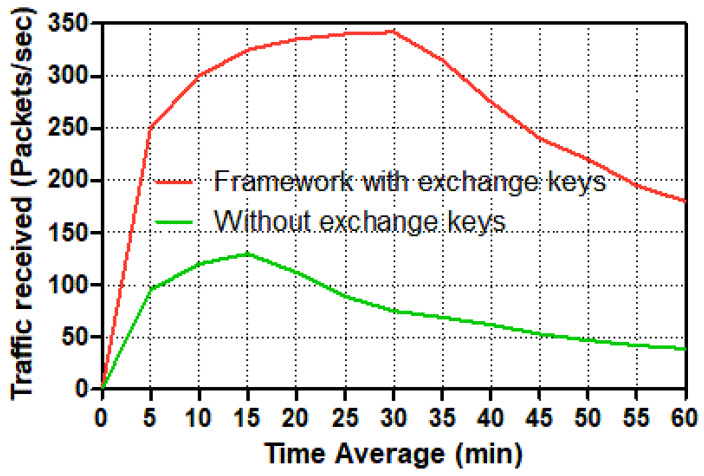
Traffic received (packets/s) in the control center for different tasks.

In this set of simulations, we evaluate the throughput packets per seconds, delay, load, and traffic received. As outlined previously, a smart meter is a two-way data transmission device. As a result, a smart meter generates packets and assigns a destination address to each one, which is subsequently sent over an Ethernet switch and then a router to the control center server. Figure 7, Figure 8, Figure 9, Figure 10, Figure 11 and Figure 12 show the point-to-point throughput packets per second, delay, load, and traffic received between a smart meter and the control center; from the smart meter to the Ethernet switch, the Ethernet switch to the router, and the router to the control center server, respectively.

Figure 7 plots the point-to-point throughput packets per second from a smart meter to an Ethernet switch. It is important to understand the difference between the two line graphs: the red line depicts secure two-way communication from a smart meter to a switch via authentication and key exchange schemes, whereas the green line depicts normal traffic without authentication and key exchange sessions as insecure two-way communication traffic. The temporal average has been used to evaluate point-to-point throughput packets per second between a smart meter and a switch in this diagram. Both line graphs began abruptly from zero to twenty mins with different amounts of packets per second; the green line for insecure traffic nearly reached seven packets per second, whereas the red line for securing traffic nearly doubled the green line, with fifteen packets per second as the point to point throughput. During the 30-min experiment, the red line had the maximum point-to-point throughput packets per second, around seventeen, while the green line had declined somewhat, to four packets per second. Following this, the red and green lines dropped drastically until they reached ten and two packets/second, respectively, at the end of our simulation time (60 mins). Overall, secure transmission employing authentication and session keys outperformed insecure traffic from a smart meter to an Ethernet switch in terms of packets per second.

Figure 8 shows that the best effort between secure and insecure two-way communication was started with a significant point of queuing delay by 0.77 s and 0.69 s, respectively. Both graphs illustrate that the number of seconds has fluctuated significantly after five mins of simulation time. After 10 mins, the seconds of queuing delay in trusted communication are expected to decrease continuously, whereas the seconds of queuing delay in un-trusted communication are expected to grow significantly. Both graphs converge significantly between 15 and 19 mins of simulation time. After that, the un-trusted traffic line (green) stayed in a steady state for about 0.73 seconds, which was almost 0.2 seconds longer than the trusted traffic line of queuing delay until the simulation period was up. To summarize, secure communication traffic between a smart meter and a switch has more service requests and key session establishment than un-secure communication traffic. When services are requested and key sessions are established, the queuing latency in trusted communication appears to be less than in un-trusted communication from a smart meter to the switch.

The load refers to how much load it takes to move packets from a smart meter to a control center or the other way around in the network. For various circumstances, Figure 9 and Figure 10 depict how much load the smart meter and control center require to transport packets across the network.

These two figures show that the number of packets per second for the trusted communication scenario in the smart meter and control center has increased dramatically. During the simulation duration, the number of packets per second peaked at around 34 mins for the smart meter and 36 mins for the control center. Because the control center sent and received packets from twenty smart meters, and the smart meter only sent and received packets from the control center, the control center load had a higher amount of packets per second than the smart meter. At the secure communication scenario in the control center, the load spiked to almost 340 packets per second after 36 mins, then dropped substantially (by roughly 50) to nearly 170 packets per second at the end. Meanwhile, the load in the unsafe scenario increased to just over 120 packets per second at 18 mins, then decreased significantly to roughly 40 packets per second at the conclusion of the simulation duration. The load in the smart meter, on the other hand, experienced a sharp increase in the number of packets per second, reaching over 17 packets at 34 mins; thereafter, the load has reduced dramatically to around 9 packets per second. The load increased dramatically from 1 to 18 mins in the insecure smart meter scenario, reaching 6 packets per second. Following that, the amount of load gradually decreased until it reached around 2 packets per second at the end of the period time. To summarize, because of the requirements of services and the establishment of keys exchange in order to connect securely, both the smart meter and the control center loaded many more packets per second in the secure scenario than in the un-secure situation. As a result, the load for trusted communication exchange was nearly double that of un-trusted communication exchange.

To evaluate the performance of receiving packets per second across the smart grid communication network, the volume of traffic received in packets per second is shown for both smart meters and control centers.

Two distinct possibilities among a smart meter and the control center server are depicted in Figure 11 and Figure 12. From 2 to 38 mins of simulation time, the traffic received packets per second among trusted communication traffic in the smart meter and the control center has increased significantly. Individually, the control center’s trusted scenario achieves a peak of around 330 packets per second at 38 mins, following which the traffic received for the trustworthy scenario reduces substantially to just under 170 packets per second at the end. Although the control center’s level rose to 120 packets per second between 2 and 19 mins in the un-trusted scenario, the receiving traffic level declined slightly to around 35 packets per second at the end. According to the smart meter, the traffic received in the trusted communication scenario spikes at roughly 38 mins, hitting 17 packets per second, before plummeting to barely 9 packets per second at the end.

Meanwhile, just before 20 mins, the number of packets received in the un-trusted communication situation increased to 6 packets per second. After 20 mins, the amount of traffic received in packets per second rapidly decreased until it reached only 2 packets per second. The control center, on the other hand, has received traffic from 20 smart meters, but according to the smart grid communication system’s design, the smart meter can only accept traffic from the control center server. Because it deals with twenty smart meters, the control center has received significantly more traffic packets per second in both scenarios, especially in the scenario with authentication and session key exchange schemes, which required a secure two-way communication channel from each smart meter to the control center. In this situation, the control center receives many more packets per second than in the other scenario, which does not use authentication or key exchange systems.

## 5. Conclusions

This paper has been enhancing and investigated the performance of securing two-way communication in smart city communication networks by using a new framework and scheme that provide an authentication and high confidentiality of over two different tasks over the OPNET simulator. In order to secure a two-way communication channel, the smart city system can achieve mutual authentication and build shared session key schemes between smart meters and the control center. We researched and assessed the security performance of the smart city communication network with and without our proposed algorithm in terms of throughput and end-to-end delay in our extensive simulation.

The results of this study reveal that raising the time average in all measures within the authentication and session key exchange schemes scenario degrades performance when compared to the situation without authentication and encryption. There is a comparison of different smart city communication network tasks to investigate authentication and session key exchange schemes in smart city communication networks, as well as other reasons for lower performance when using authentication and session key exchange schemes in smart city communication networks.

In the future, instead of considering a handshaking algorithm between neighborhoods, it would be interesting to increase and enhance the cyber security systems by using the proposed framework for underwater sensor networks in [27,28,29]. Another area of future interest is verifying the protocol’s reliability when employing the mobile Autonomous Underwater Vehicle (AUV) in a distributed way in order to develop an efficient cyber security framework [30,31,32,33].

## Figures and Tables

**Table 1:** The phases’ configuration.

Task Name	Phase Name	Start After	Source	Destination
Authentication task	Smart Meter to Control Center	Application Starts	Smart Meter	Control Center
	Verify Control Centre	Previous Phase Ends	Smart Meter	Not Applicable
Encryption task	Smart Meter to Control Center	Application Starts	Smart Meter	Control Center
	Control Center to Smart Meter	Application Starts	Control Center	Smart Meter

**Table 2:** Simulation parameters.

Parameter	Value
Initialization time of the first phase	0 s
Request count	1 (constant)
Request packet size	3 Kbytes (constant)
Processing time	0.5 s
Number of replies	1 (constant)
Request packet size of the response	5 kbytes (constant)
Initialization time of the second phase	0.2 s
Request count of the second phase	0 (constant)
Initialization time of encryption task	0.2 s
Request count of encryption task	1000 (constant)
Inter-request time	1.2 s (exponential)
Request packet size of encryption task	1–10 kbytes (uniform_int)
Simulation time for each run	3600 s

## Data Availability

Not applicable.
